# Twenty-year trajectories of alcohol consumption during midlife and atherosclerotic thickening in early old age: findings from two British population cohort studies

**DOI:** 10.1186/s12916-016-0656-9

**Published:** 2016-07-29

**Authors:** Annie Britton, Rebecca Hardy, Diana Kuh, John Deanfield, Marietta Charakida, Steven Bell

**Affiliations:** 1 Research Department of Epidemiology and Public Health, University College London, 1-19 Torrington Place, London, WC1E 6BT UK; 2 MRC Unit for Lifelong Health & Ageing at University College London, London, UK; 3 National Centre for Cardiovascular Prevention and Outcomes, UCL, London, UK

**Keywords:** Alcohol, Life course, Longitudinal, Atherosclerosis

## Abstract

**Background:**

Epidemiological evidence indicates a protective effect of light-moderate drinking on cardiovascular disease and an increased risk for heavier drinking. Nevertheless, the effect of alcohol on atherosclerotic changes in vessel walls is disputed. Most previous studies have only looked at the cross-sectional relationship between alcohol and carotid intima media thickness (cIMT) – a surrogate marker of atherosclerosis. Single measurements of alcohol assume that alcohol exposure is stable and ignore the possible cumulative effects of harm, leading to possibly incorrect inferences.

**Methods:**

Data were retrieved from two UK population based cohort studies: the Whitehall II cohort of civil servants and the MRC National Survey of Health and Development (combined sample size of 5403 men and women). Twenty year-drinking trajectories during midlife were linked to measures of cIMT when participants were in early old age, and adjusted for age, sex, socioeconomic position, ethnicity and smoking.

**Results:**

Those who consistently drank heavily had an increased cIMT compared to stable moderate drinkers (pooled difference in cIMT 0.021 mm; 95 % CI 0.002 to 0.039), after adjustment for covariates. This was not detected in cross-sectional analyses. Former drinkers also had an increased cIMT compared to moderate drinkers (pooled difference in cIMT 0.021; 95 % CI 0.005 to 0.037). There were no appreciable differences in cIMT between non-drinkers and consistent moderate drinkers.

**Conclusion:**

The drinking habits among adults during midlife affect the atherosclerotic process and sustained heavy drinking is associated with an increased cIMT compared to stable moderate drinkers. This finding was not seen when only using cross-sectional analyses, thus highlighting the importance of taking a life course approach. There was no evidence of a favourable atherosclerotic profile from stable moderate drinking compared to stable non-drinking.

**Electronic supplementary material:**

The online version of this article (doi:10.1186/s12916-016-0656-9) contains supplementary material, which is available to authorized users.

## Background

Alcohol consumption is one of the biggest public health challenges facing modern society and is ranked as the world’s third largest risk factor for disease burden [1]. There are also currently concerns about the long-term ill health effects of the drinking habits of middle-aged adults, a group who have been described as ‘hidden’ risky drinkers [2].

Epidemiological evidence indicates a protective effect of light-moderate alcohol consumption on cardiovascular morbidity and mortality compared to non-drinking and an increased risk of cardiovascular events amongst heavier drinkers [3–5]. The endpoint of cardiovascular disease (CVD) often occurs later in life; the extent to which alcohol consumption earlier in the life course is implicated in the process is less clear. As yet, the biological mechanisms underlying the association between alcohol intake and CVD are not fully elucidated, and it has been argued that the protective effect observed in observational studies may, in part, be due to misclassification errors and reporting biases [6]. If the cardiovascular benefits of moderate alcohol consumption are genuine, they may be explained in part by alcohol’s influence on serum lipids [7] and other cardiovascular biomarkers [8], blood pressure, and atherosclerosis [9]. Nevertheless, the effect of alcohol on atherosclerotic changes in vessel walls is disputed. Carotid intima media thickness (cIMT) is an intermediate phenotype of early atherosclerosis or a marker of subclinical organ damage that independently predicts vascular events [10]. Some studies have found evidence of a reduced risk of atherosclerosis (as measured by cIMT) in moderate drinkers [11–17], while others report no association [18–21], or an increased risk [22–24]. Most of the previous studies have only looked at the cross-sectional association between alcohol intake and cIMT. Single observations of alcohol consumption assume that drinking is a stable behaviour. However, there is evidence from descriptive studies that individuals change their alcohol consumption levels over time [25, 26]. Research on the health consequences of alcohol, therefore, needs to address the effects of changes in drinking behaviour over the life course [27]. Failure to include such dynamics can lead to incorrect inferences about the effects of alcohol on chronic disease risk [28].

In this paper we sought to (1) describe trajectories of alcohol consumption during midlife, (2) link these trajectories to risk of atherosclerosis, as measured by cIMT in early old-age, and (3) compare these associations with cross-sectional findings in the same cohorts.

## Methods

Data were drawn from two UK population-based cohorts: the Whitehall II Cohort of British Civil Servants and the MRC National Survey of Health and Development (NSHD). In both cohorts, alcohol consumption was self-reported at four time points over a 20-year period and cIMT was measured when participants were in early old-age (50–74 years).

### Whitehall II study

The Whitehall II study was established in 1985 as a longitudinal study to examine the socioeconomic gradient in health and disease among 10,308 civil servants (6895 men and 3413 women) [29]. All civil servants aged 35–55 years in 20 London-based departments were invited to participate by letter and 73 % agreed. Baseline examination (Phase 1) took place during 1985–1988, and involved a clinical examination and a self-administered questionnaire containing sections on demographic characteristics, health, lifestyle factors, work characteristics, social support and life events. The clinical examination included measures of blood pressure, anthropometry, biochemical measurements, neuroendocrine function, and subclinical markers of cardiovascular disease. Subsequent phases of data collection have alternated between postal questionnaire alone and postal questionnaire accompanied by a clinical examination. Data used in the analyses came from phases 1 (1985–1988), 3 (1991–1994), 5 (1997–1999) and 7 (2002–2004) of the study.

From the 10,308 participants at baseline, 3321 did not participate in phase 7 (584 died, 2757 withdrew or did not respond). Of the eligible 6967, 4109 (59 %) had a valid measure of cIMT. A further 49 people were missing age or alcohol data and 20 were missing information on socioeconomic position (SEP) and/or smoking.

The University College London Medical School Committee on the ethics of human research approved the Whitehall II study. Written informed consent was obtained at baseline and renewed at each contact. Whitehall II data, protocols and other metadata are available to bona fide researchers for research purposes (the data sharing policy is available at http://www.ucl.ac.uk/whitehallII/data-sharing).

### NSHD

The NSHD is a nationally representative sample of 5362 singleton births to married parents in England, Scotland and Wales, stratified by social class in 1 week in March 1946 in Britain. The sample has been followed-up 24 times since birth to age 60–64 years [30]; the 25^th^ follow-up at 68–69 years is ongoing. The study protocol received ethical approval from the Central Manchester Research Ethics Committee for a clinic data collection taking place in Manchester, Birmingham, Cardiff and London at 60–64 years. Ethical permission was given by the Scotland A Research Ethics Committee for the data collection taking place in Edinburgh. Written informed consent was obtained from the study member at each stage of data collection. Bona fide researchers can apply to access the NSHD data via a standard application procedure (further details available at: http://www.nshd.mrc.ac.uk/data.aspx).

At the clinical follow-up, when study members were aged between 60 and 64 years, 2856 of those still alive and with a known current address in mainland Britain were invited for assessment at one of six clinical research facilities; those unable or unwilling to travel were offered a home visit by a research nurse [30]. Of those invited, a total 2229 participants (78 %) underwent assessment: 1690 attended a clinical research facility and the remaining 539 were seen in their homes [31]; 1532 participants had a valid measure of cIMT (62 % of eligible). A further 151 had missing alcohol or age and 131 missing information on SEP and/or smoking.

Therefore, our analyses are based on 4060 Whitehall II participants and 1391 NSHD participants with valid cIMT and repeat alcohol data. We used modified Poisson regression [32] to compare baseline characteristics of those excluded and included in the analysis, and found those included were more likely to be of higher SEP, moderate drinkers and never smokers.

### Alcohol consumption

In both cohorts, alcohol measurements were available at four time points over a 20-year period before the measurement of cIMT, at roughly comparable ages. In Whitehall II, at phases 1, 3, 5 and 7, participants were asked to report the number of alcoholic drinks they had consumed in the last 7 days. Drinks were converted into UK units of alcohol (whereby one unit is equivalent to 8 g of ethanol) using a conservative estimate of one UK unit for each measure of spirits and glass of wine, and two UK units for each pint of beer. These converted measurements were then summed to define the total weekly number of UK units consumed.

In NSHD, at ages 36, 43, 53 and 60–64, alcohol consumption was assessed using a 5-day food diary. From these diaries, an estimate was derived of total alcohol consumed per week in UK units. Further details on harmonisation of alcohol consumption in these cohorts can be found elsewhere [26].

Categories of alcohol consumption were then created based on existing UK guidelines for sensible drinking at the time of data collection [33], these were: none, “moderate” (within guidelines (1–14 [8–112 g] units per week for women, 1–21 [8–168 g] units per week for men)), and “heavy” (above guidelines (15+ units for women, 22+ units for men)). Trajectories of alcohol consumption over the four measurement periods were then classified as (1) stable none, (2) stable moderate, (3) stable heavy, (4) mostly moderate (majority of phases were moderate), and (5) mostly heavy or as (6) former drinkers (previously reported consumption but none in the most recent phase). When an individual reported moderate and heavy on an equal number of occasions, participants were assigned to the mostly heavy drinking group. Individuals drinking at the cIMT assessment phase but not at other occasions were classified as mostly moderate or heavy on the basis of their drinking at the time. Participants were permitted one missing alcohol value during follow-up.

### Carotid IMT

At phase 7 in the Whitehall II study, when participants were aged 50–74 years old (mean age 61 years), ultrasound vascular measures were performed at the Vascular Physiology Unit, Institute of Child Health, London, UK. Measurements were taken using the Aloka 5500 with a 7.5 MHz transducer. IMT was measured in the right and left common carotid arteries. Longitudinal images of the common carotid artery, triggered on the R-wave of the ECG, were magnified and recorded in DICOM format as a cine-loop on the hard drive of the ultrasound machine for later analysis. The common carotid IMT was measured at its thickest part 1 cm proximal to the bifurcation. A measurement was taken between the leading edge of the intima and the media adventitia on three separate images on each side using electronic callipers and the mean of the six measures was used for analysis.

At age 60–64 years in NSHD, cIMT was imaged longitudinally in the right and left common carotid arteries, 1 cm proximal to the bifurcation. Measures were made with the Vivid I ultrasound scanner (GE Healthcare; Chalfont St Giles, UK) with a high resolution probe (12 MHz). Ten second cine-loops were recorded in DICOM format and downloaded for offline analysis. Analysis of the cine-loops was performed at the same Vascular Physiology Unit as for the Whitehall II study. Three end-diastolic frames from each lateral view were selected and the mean of the six measures used for analysis.

### Covariates

Covariates included age, sex, ethnicity, smoking status and SEP. Smoking status (never, ex, current 0–10, 11+ cigarettes per day) and SEP (defined using last known employment grade as high, intermediate and low in Whitehall II and based on last known registrar general social class in NSHD – categories 1 and 2: highest; 3 and 4: intermediate; 5 and 6: lowest) were identified from self-reported questionnaires completed at the time of cIMT measurement. Ethnicity was only available for the Whitehall II cohort (white and non-white). The NSHD participants are all white as they were born in Britain before the start of mass immigration into the country.

### Analysis

Quantile regression was used for the analysis as cIMT measurements were mildly skewed [34]. In preliminary analyses we found no significant effect modification by sex so data were therefore pooled and adjustment made for sex. We performed two stages of adjustment, firstly for age and sex and secondly for age, sex, SEP, smoking and ethnicity (in Whitehall II). We performed analyses separately for each cohort and then combined estimates from each study using random-effects meta-analysis.

Those with existing coronary heart disease (CHD) (validated events) and/or type 2 diabetes (cases defined using oral glucose tolerance tests, HbA1c values and/or use of diabetes medication) were excluded in sensitivity analyses using Whitehall II data (*n* = 1,501) to assess the potential impact on overall findings if those with known disease had changed their alcohol consumption in response to disease onset. All analyses were performed using Stata 14 from August to September 2015.

## Results

The most common derived alcohol trajectories were “stable moderate” (39.8 % Whitehall, 34.4 % NSHD) and “mostly moderate” (24.9 % Whitehall, 31.3 % NSHD) (Table 1). Smaller groups were “stable none” (5.3 % Whitehall II, 4.5 % NSHD) and “stable heavy” (6.8 % Whitehall II, 3.5 % NSHD). “Stable heavy” drinkers were more likely to be male, current smokers and of white ethnicity (in Whitehall II) (Table 1). “Stable none” drinkers were more likely to be female, non-smokers, of lower SEP and non-white. The median cIMT level measured in the overall Whitehall participant cohort was 0.77 mm (IQR 0.68–0.87), and 0.77 mm (IQR 0.68–0.88) for men and 0.77 mm (IQR 0.68–0.85) for women. The median lateral view cIMT was 0.67 mm (IQR 0.60–0.76) in the NSHD overall cohort, and 0.68 mm (IQR 0.61–0.79) for men and 0.66 mm (IQR 0.59–0.73) for women.

**Table 1:** Characteristics of participants by alcohol trajectories in Whitehall II and NSHD

			Alcohol trajectories over previous 20 years				Total
	Stable none (*n*, %)	Stable moderate drinker (*n*, %)	Stable heavy drinker (*n*, %)	Mostly moderate (*n*, %)	Mostly heavy (*n*, %)	Former drinker (*n*, %)	
Whitehall II Total	216 (5.3)	1615 (39.8)	274 (6.8)	1010 (24.9)	536 (13.2)	409 (10.1)	4060 (100)
Mean age, years (SD)	61.6 (6.3)	61.4 (5.9)	60.0 (5.4)	60.8 (5.8)	60.7 (5.79)	61.5 (6.0)	61.1 (5.9)
Median cIMT, mm (IQR)	0.78 (0.70–0.90)	0.77 (0.68–0.87)	0.77 (0.70–0.88)	0.77 (0.68–0.87)	0.87 (0.70–0.90	0.78 (0.70–0.87)	0.77 (0.68–0.87)
Male	96 (44.4)	1234 (76.4)	233 (85)	703 (69.6)	437 (81.5)	211 (51.6)	2914 (71.8)
Female	120 (55.5)	381 (23.6)	41 (15)	307 (30.4)	99 (18.5)	198 (48.4)	1146 (28.2)
Smoking: Never	155 (72.1)	883 (54.8)	87 (31.8)	501 (50.9)	198 (37.1)	210 (51.6)	2043 (50.6)
Ex-smoker	47 (21.9)	646 (40.1)	145 (52.9)	429 (42.9)	294 (55.2)	159 (39.1)	1720 (42.6)
Current 1–10 cpd	8 (3.7)	46 (2.9)	14 (5.1)	21 (2.1)	16 (3.0)	21 (5.2)	126 (3.1)
Current 11+ cpd	5 (2.3)	35 (2.2)	28 (10.2)	41 (4.1)	25 (4.7)	17 (4.2)	151 (3.7)
SEP: High	35 (16.2)	858 (53.1)	159 (58.0)	441 (43.7)	302 (56.3)	93 (22.7)	1881 (46.3)
Intermediate	107 (49.5)	648 (40.1)	111 (40.5)	473 (46.8)	215 (40.1)	210 (51.3)	1768 (43.5)
Low	74 (34.3)	109 (6.7)	4 (1.5)	96 (9.5)	19 (3.5)	106 (25.9)	411 (10.1)
White	132 (61.1)	1516 (93.9)	270 (98.5)	934 (92.5)	522 (97.4)	340 (83.1)	3715 (91.5)
Non-white	84 (38.9)	99 (6.1)	4 (1.5)	76 (7.5)	14 (2.6)	69 (16.9)	342 (8.5)
NSHD Total	62 (4.5)	478 (34.4)	49 (3.5)	436 (31.3)	181 (13.0)	185 (13.3)	1391 (100)
Mean age, years (SD)	63.2 (1.03)	63.3 (1.12)	63.2 (1.17)	63.2 (1.16)	63.2 (1.20)	63.4 (0.99)	63.3 (1.12)
Median cIMT, mm (IQR)	0.63 (0.57–0.76)	0.66 (0.60–0.75)	0.68 (0.62–0.80)	0.67 (0.59–0.75)	0.67 (0.59–0.77)	0.68 (0.61–0.76)	0.67 (0.60–0.76)
Male	16 (25.8)	230 (48.1)	43 (87.8)	207 (47.5)	112 (61.9)	65 (35.1)	673 (48.4)
Female	46 (74.2)	248 (51.9)	6 (12.2)	229 (52.5)	69 (38.1)	120 (64.9)	718 (51.6)
Smoking: Never	31 (55.4)	180 (40.2)	4 (9.3)	129 (31.8)	38 (22.4)	59 (34.9)	441 (34.1)
Ex-smoker	19 (33.9)	242 (54.0)	33 (76.7)	248 (61.1)	106 (62.4)	92 (54.4)	740 (57.3)
Current 1–10 cpd	5 (8.9)	11 (2.5)	1 (2.3)	8 (2.0)	9 (5.3)	6 (3.6)	40 (3.1)
Current 11+ cpd	1 (1.8)	15 (3.3)	5 (11.6)	21 (5.2)	17 (10.0)	12 (7.1)	71 (5.5)
SEP: High	20 (36.4)	263 (59.2)	26 (57.8)	227 (56.8)	104 (61.9)	73 (44.5)	713 (55.9)
Intermediate	21 (38.2)	158 (35.6)	14 (31.1)	131 (32.8)	52 (31.0)	70 (42.7)	446 (35.0)
Low	55 (25.5)	23 (5.2)	5 (11.1)	42 (10.5)	12 (7.1)	21 (12.8)	117 (9.2)

*SEP* Socioeconomic position, *cIMT* Carotid intima media thickness, *SD* Standard Deviation, *IQR* Interquartile range, *cpd* Cigarettes per day

**Fig. 1:**
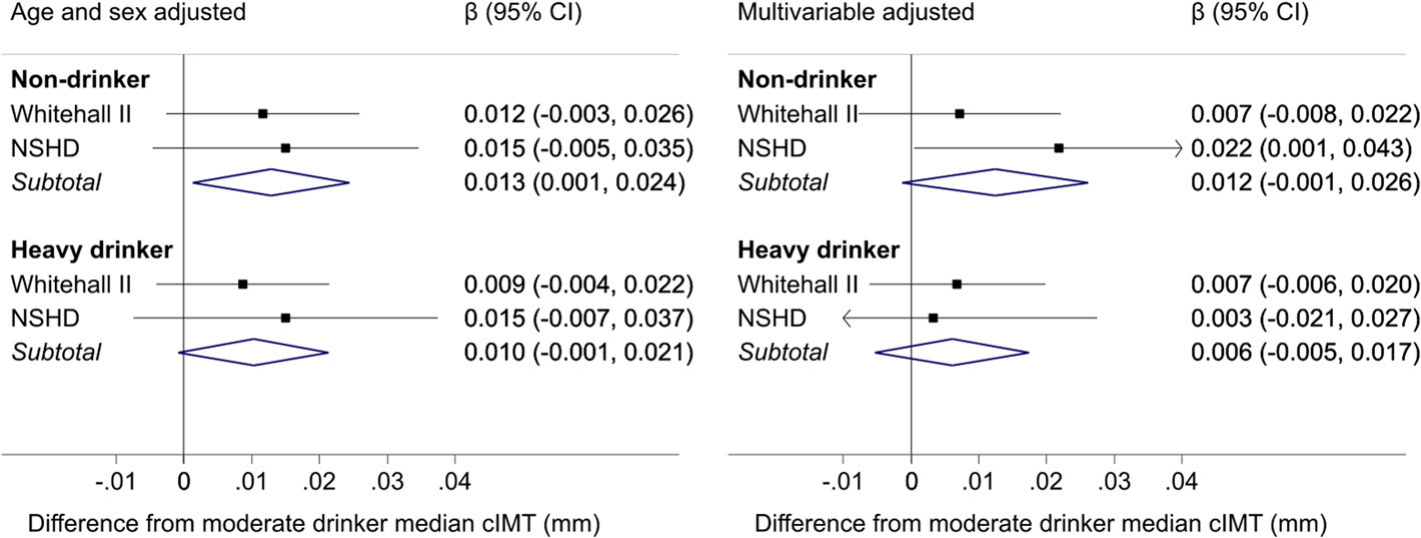
Meta-analysis of the cross-sectional difference in carotid intima media thickness (mm) by current alcohol consumption category (reference group moderate drinkers). Multivariables adjusted = age, sex, ethnicity (in Whitehall II), socioeconomic position and smoking status

Cross-sectional analyses showed slightly elevated cIMT values amongst non-drinkers (pooled β = 0.012 mm, 95 % CI –0.001 to 0.026) compared to moderate drinkers (Fig. 1). When non-drinkers were separated into former drinkers and never drinkers, the cIMT was higher in former drinkers than never drinkers (available in Additional file 1: Figure S1). In cross-sectional analyses there was little difference in atherosclerotic thickening between heavy and moderate drinkers (pooled β = 0.006 mm, 95 % CI –0.005 to 0.017) (Fig. 1).

**Fig. 2:**
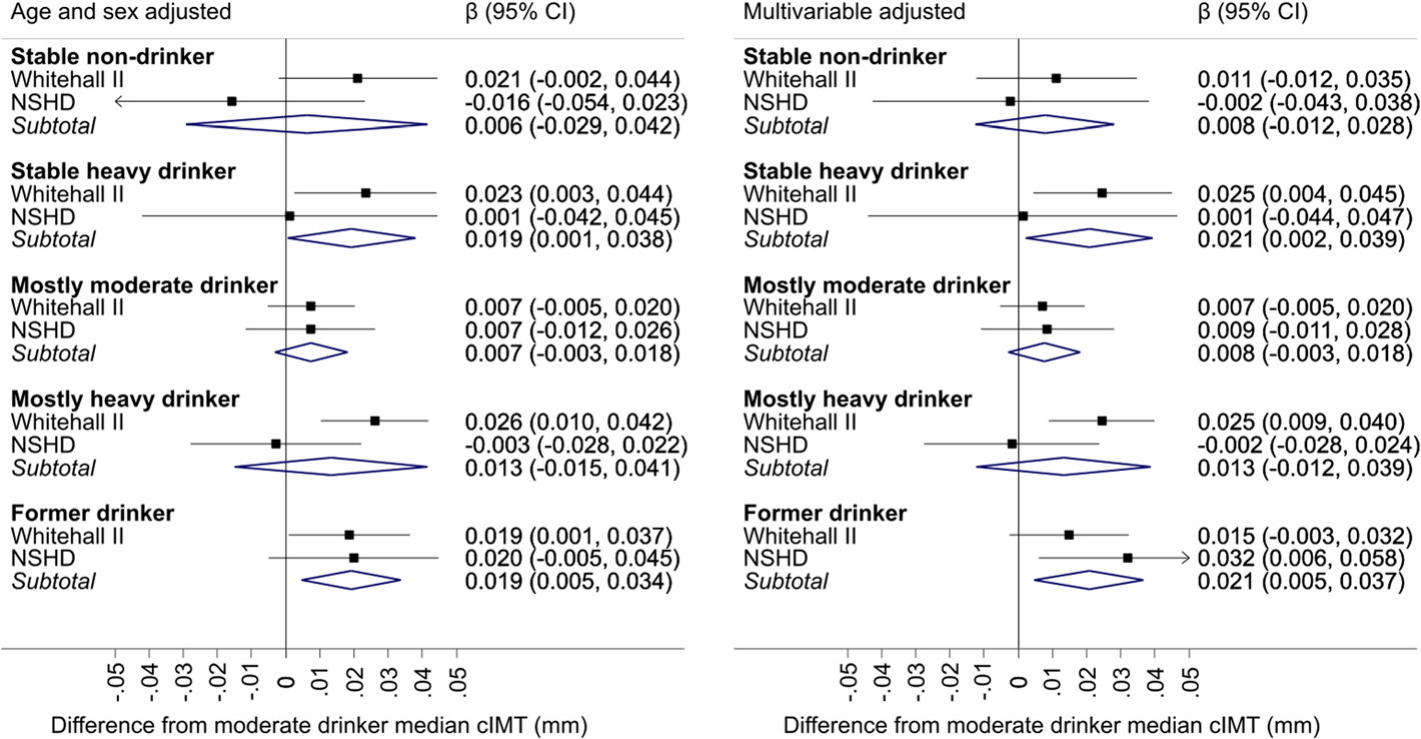
Meta-analysis of difference in carotid intima media thickness (mm) by 20-year trajectories of alcohol consumption (reference stable moderate drinkers). Multivariables adjusted = age, sex, ethnicity (in Whitehall II), socioeconomic position and smoking status

The association between trajectories of drinking during midlife and cIMT are shown in Fig. 2. Full results from regression analyses are shown in Additional file 2: Table S1 and S2. “Stable heavy” drinkers over the 20-year measurement period had elevated cIMT compared to “stable moderate” drinkers (pooled difference of 0.021 mm, 95 % CI 0.002 to 0.039), after adjustment for covariates. There were no appreciable differences between “stable non-drinkers” and “stable moderate” drinkers (pooled β = 0.008 mm, 95 % CI –0.003 to 0.018). “Former drinkers” had raised cIMT (pooled β = 0.021 mm, 95 % CI 0.005 to 0.037). Mostly moderate and mostly heavy were similar. Excluding those with prevalent CHD/diabetes in the Whitehall II cohort did not substantially alter the estimates (Additional file 1: Figure S2).

## Discussion

We derived 20-year drinking trajectories during midlife in two separate British population-based cohort studies and linked these to cIMT in early old age. We found evidence to suggest that former drinkers and those with sustained heavy drinking during midlife had greater cIMT values than stable moderate drinkers. We also observed that stable moderate drinkers did not have reduced cIMT values compared to stable non-drinkers. Combined, these findings indicate that midlife drinking habits affect the atherosclerotic process.

The risks associated with heavy drinking in midlife were only appreciable when considering a 20-year trajectory of drinking and not in cross-sectional analyses. This highlights the need to take a life course approach and our study should be replicated with other outcomes. Our data start from around age 35 years. Others have suggested that an adverse effect on atherogenesis from drinking may occur even earlier in life. In a cohort of young Finns, aged 24–39 years, there was a dose–response relationship between alcohol consumption and increased cIMT [23].

However, there is a risk of misclassification if only one measure of alcohol is considered. For example, we found that, when current non-drinkers were separated into former drinkers and never drinkers, the median cIMT levels were substantially higher in the former drinkers. Former drinkers may include individuals who experienced ill-health (including increased vascular problems as indicated by higher cIMT) and subsequently quit. This concurs with the well-known ‘sick-quitter’ phenomenon whereby former drinkers who become ill and cease drinking are classified as non-drinkers and this can erroneously lead to suggested protective effects of drinking compared to not drinking [35]. Alcohol consumption is not a stable phenomenon over the life course. Exposure varies, as shown in this paper by the large proportion in both cohorts who did not have stable drinking trajectories over the 20 years, and, elsewhere, we have shown how mean consumption and frequency changes in these cohorts and others [26].

Much of the existing literature uses data with alcohol exposure measured at a single time-point and takes a mixed group of “non-drinkers” as the reference group. Ours is the first study to look at trajectories of alcohol and risk of cIMT, which makes drawing comparisons between our work and others difficult. Our cross-sectional findings concur with those from the Atherosclerosis Risk in Communities study and the National Heart, Lung, and Blood Institute Family Heart Study, which indicate no association between current alcohol intake and carotid artery wall thickness [18, 19]. Likewise, data from over 6000 men and women in three French cities showed no marked relationship of alcohol and cIMT [20]. Others have found a U-shaped relationship between alcohol and cIMT in cross-sectional analyses. In the Cardiovascular Health Study investigating subjects over 65 years, consumers of 1–6 drinks per week (equalling < 15 g/d) had a cIMT 0.07 mm lower than abstainers, whereas consumers of 14 or more drinks (equalling > 30 g/d) had an IMT 0.07 mm higher than abstainers. Like in the present study, they found that former drinkers had an increased cIMT [16].

Our trajectory work suggests that clinicians need to place emphasis on drinking histories as well as current drinking behaviour. Attempting to quantify the effect of heavy drinking on atherosclerosis is challenging. Elsewhere, it has been shown, using data from the Whitehall II study, that the mean progression rate of cIMT is estimated to be 0.012 ± 0.028 mm per year [36]. Therefore, the effects of consistent heavy drinking roughly equate to 19 months expedited vascular ageing.

Measuring alcohol use over time is likely to be more aetiologically relevant than a snap shot of alcohol at one point in time. For example, it is reasonable to hypothesise that sustained high levels of drinking increase the formulation of atheromas on the vessel wall over time [23, 24].

A major strength of this study is our ability to use repeated measures of alcohol consumption on the same individuals over two decades before the measurement of cIMT. The derived trajectories have policy relevance as they are defined by UK government guidelines [33]. Treating cIMT as a surrogate endpoint allowed for us to investigate midlife drinking as a risk factor for early atherosclerosis. This enabled us to determine how drinking during this period might set the stage for the development of CVD, before it is necessarily symptomatic.

Our study is limited in that alcohol consumption was self-reported and therefore at risk of over and under-reporting [37, 38]. We were only able to capture snap shots of drinking over the past week/5 days and have assumed that these are a general representation of levels consumed over that period. This may introduce error, but we utilised the repetition of these snap shops to more accurately create trajectories than simple use of a baseline measure. Our sample is made up of individuals who have remained in an epidemiological study for decades. This is a form of selection bias [39] and may mean that our sample is no longer representative of the original population from which it was drawn. We found those remaining in the studies and attending clinical research facilities to be a healthier subsample than those who dropped out or who did not participate fully. Furthermore, population-based studies do not capture the extremes of drinking and therefore may be underpowered to look at the effects of very heavy drinking. At the time of cIMT measurement, the proportion drinking in excess of guidelines was 19.3 % in Whitehall II (21.9 % men and 12.8 % women) and 13.0 % in NSHD (17.8 % men and 7.9 % women), which is considerably lower than recent estimates from Health Survey for England (31 % men and 20 % women aged 55–64 years) [40]. It may be that NSHD, in particular, was underpowered to detect effects among sustained heavy drinkers (who constituted only 3.5 % of the sample). Furthermore, we were unable to assess the effects of binge drinking as these data were not adequately captured in both studies at that time. Although we included several covariates in the analysis, as with most observational studies, we cannot rule out that possibility of residual confounding.

Future work should consider the progression of cIMT [18] into older age and whether changes in atherosclerosis, and ultimately CHD cases, are affected by changes in drinking levels. Furthermore, it would be interesting to extend the work on pattern of consumption [24] to see whether sustained ‘binge’ drinking confers an increased risk of higher cIMT compared to regular heavy drinking [41], as is found for CHD overall [42].

## Conclusions

These findings indicate that the drinking habits adopted by adults during midlife affect the atherosclerotic process, and that sustained heavy drinking is associated with an increased risk of poor cardiovascular health compared to stable moderate drinkers. This finding was not seen when using cross-sectional analyses only, thus highlighting the importance of taking a longitudinal approach. Furthermore, there was no evidence of a favourable atherosclerotic profile among stable moderate drinkers compared to non-drinkers.

## Abbreviations

cIMT, carotid intima media thickness; CVD, cardiovascular disease; NSHD, National Survey of Health and Development; SEP, socioeconomic position

## Additional files

Additional file 1: Figure S1. Meta-analysis of current drinking separated into “former drinker” and “never drinker” categories (reference moderate drinkers). Figure S2 Derived trajectories of alcohol and difference in carotid intima media thickness (cIMT, mm) in those with and without prevalent CHD/diabetes in the Whitehall II study. (DOCX 428 KB) Additional file 2: Table S1. Cross sectional differences in cIMT (mm) by current alcohol consumption category (reference group moderate drinkers). Full results from regression models Table S2: Meta-analysis of difference in cIMT (mm) by 20 year trajectory of alcohol consumption (reference stable moderate drinkers). Full results from regression models. (DOCX 16 KB)
