# Supplementary material for: Twenty-year trajectories of alcohol consumption during midlife and atherosclerotic thickening in early old age: findings from two British population cohort studies
Source: BMC Med. 2016 Jul 29;14:111. doi: 10.1186/s12916-016-0656-9 (PMC4967336; doi:10.1186/s12916-016-0656-9)

Figure S1. Meta-analysis of current drinking separated into former drinker and never drinker category (reference moderate drinkers)


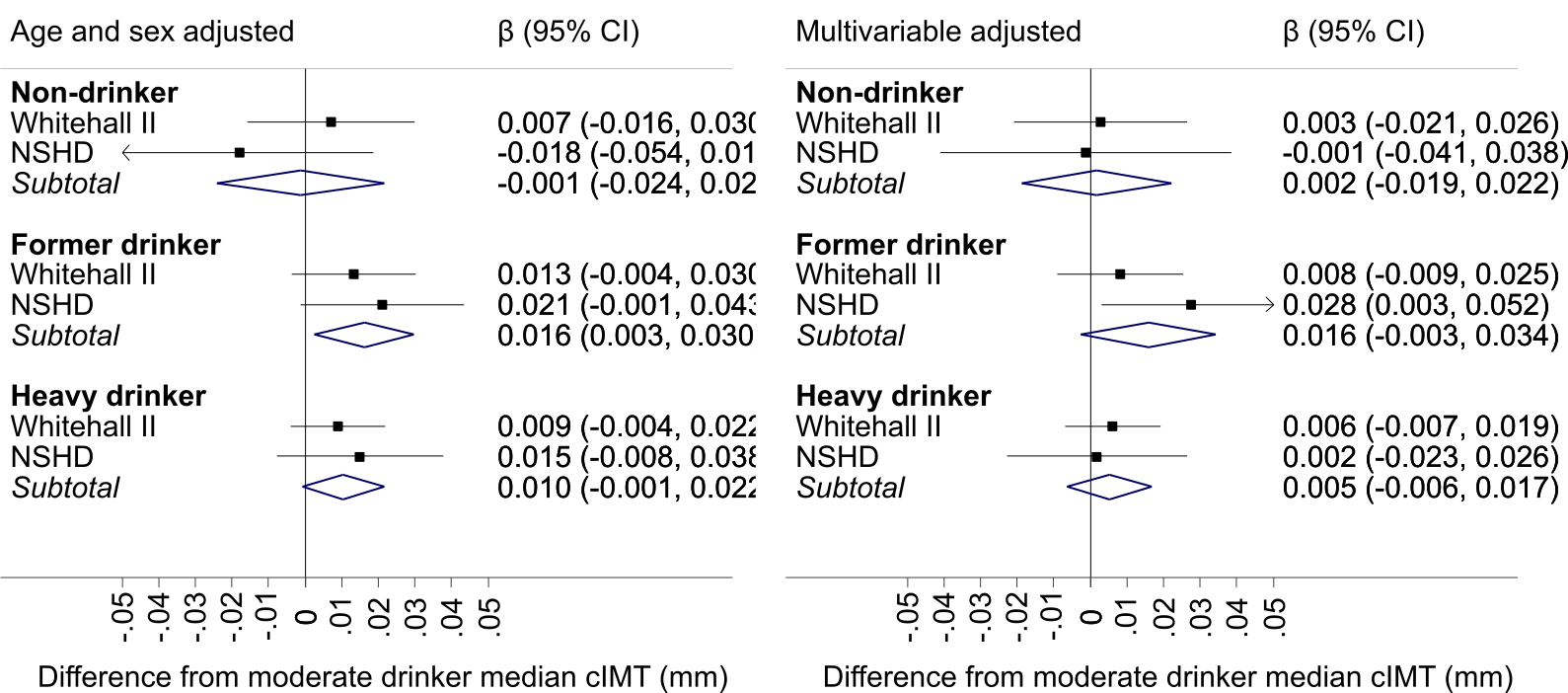


Multivariable adjusted = age, sex, ethnicity (in Whitehall II), socio-economic position and smoking status

Figure S2. Derived trajectories of alcohol and difference in cIMT (mm) in those with and without prevalent CHD/diabetes in the Whitehall II study


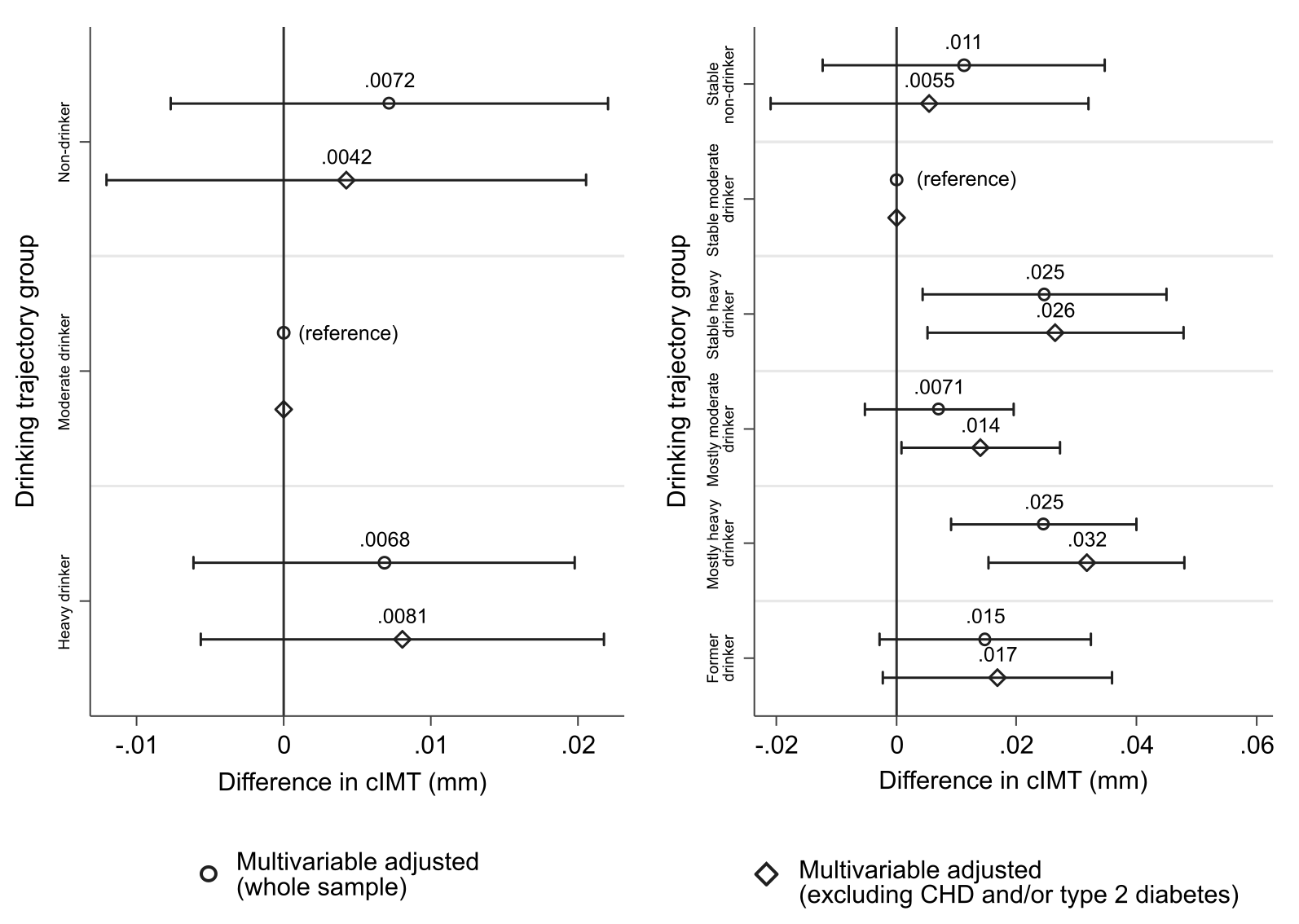

Supplement: Additional file 1: Figure S1. — Meta-analysis of current drinking separated into “former drinker” and “never drinker” categories (reference moderate drinkers). Figure S2 Derived trajectories of alcohol and difference in carotid intima media thickness (cIMT, mm) in those with and without prevalent CHD/diabetes in the Whitehall II study. (DOCX 428 KB) [file 12916_2016_656_MOESM1_ESM.docx]
