# Supplementary material for: Twenty-year trajectories of alcohol consumption during midlife and atherosclerotic thickening in early old age: findings from two British population cohort studies
Source: BMC Med. 2016 Jul 29;14:111. doi: 10.1186/s12916-016-0656-9 (PMC4967336; doi:10.1186/s12916-016-0656-9)
Supplement: Additional file 2: Table S1. — Cross sectional differences in cIMT (mm) by current alcohol consumption category (reference group moderate drinkers). Full results from regression models Table S2: Meta-analysis of difference in cIMT (mm) by 20 year trajectory of alcohol consumption (reference stable moderate drinkers). Full results from regression models. (DOCX 16 KB) [file 12916_2016_656_MOESM2_ESM.docx]

Table S1. Cross sectional differences in cIMT (mm) by current alcohol consumption category (reference group moderate drinkers). Full results from regression models

|  | Whitehall II | Whitehall II | NSHD | NSHD |
| --- | --- | --- | --- | --- |
|  | cIMT (age and sex) | cIMT (multivariable adjusted) | cIMT (age and sex) | cIMT (multivariable adjusted) |
| None | 0.012 [-0.003,0.026] | 0.007 [-0.008,0.022] | 0.015 [-0.005,0.035] | 0.022* [0.001,0.043] |
| Moderate | 0.000 [ref] | 0.000 [ref] | 0.000 [ref] | 0.000 [ref] |
| Heavy | 0.009 [-0.004,0.022] | 0.007 [-0.006,0.020] | 0.015 [-0.007,0.037] | 0.003 [-0.021,0.027] |
| Age | 0.007*** [0.006,0.008] | 0.007*** [0.006,0.008] | 0.007* [0.000,0.013] | 0.005 [-0.002,0.012] |
| Sex | -0.014* [-0.025,-0.003] | -0.014* [-0.026,-0.002] | -0.027*** [-0.042,-0.012] | -0.020* [-0.036,-0.004] |
| White |  | 0.000 [ref] |  |  |
| Non-White |  | 0.040*** [0.021,0.059] |  |  |
| SEP - High |  | 0.000 [ref] |  | 0.000 [ref] |
| Intermediate |  | -0.001 [-0.012,0.010] |  | -0.002 [-0.019,0.015] |
| Low |  | -0.006 [-0.026,0.014] |  | -0.017 [-0.046,0.011] |
| Never smoker |  | 0.000 [ref] |  | 0.000 [ref] |
| Ex-smoker |  | 0.016** [0.006,0.027] |  | 0.013 [-0.004,0.030] |
| Current 1-10 |  | 0.039** [0.010,0.068] |  | 0.021 [-0.026,0.068] |
| Current 11+ |  | 0.015 [-0.012,0.042] |  | 0.041* [0.004,0.077] |
| Constant | 0.766*** [0.759,0.773] | 0.757*** [0.748,0.767] | 0.657*** [0.632,0.681] | 0.648*** [0.619,0.677] |
| Observations | 4021 | 4011 | 1381 | 1252 |

95% confidence intervals in brackets

* p<0.05, ** p<0.01, *** p<0.001

Table S2. Meta-analysis of difference in CIMT (mm) by 20 year trajectories of alcohol consumption (reference stable moderate drinkers). Full results from regression models

|  | Whitehall II | Whitehall II | NSHD | NSHD |
| --- | --- | --- | --- | --- |
|  | cIMT (age and sex) | cIMT (multivariable adjusted) | cIMT (age and sex) | cIMT (multivariable adjusted) |
| Stable non-drinker | 0.021 [-0.002,0.044] | 0.011 [-0.012,0.035] | -0.016 [-0.054,0.023] | -0.002 [-0.043,0.038] |
| Stable moderate | 0.000 [ref] | 0.000 [ref] | 0.000 [ref] | 0.000 [ref] |
| Stable heavy | 0.023* [0.003,0.044] | 0.025* [0.004,0.045] | 0.001 [-0.042,0.045] | 0.001 [-0.044,0.047] |
| Mostly moderate | 0.007 [-0.005,0.020] | 0.007 [-0.005,0.020] | 0.007 [-0.012,0.026] | 0.009 [-0.011,0.028] |
| Mostly heavy | 0.026** [0.010,0.042] | 0.025** [0.009,0.040] | -0.003 [-0.028,0.022] | -0.002 [-0.028,0.024] |
| Former drinker | 0.019* [0.001,0.037] | 0.015 [-0.003,0.032] | 0.020 [-0.005,0.045] | 0.032* [0.006,0.058] |
| Age | 0.007*** [0.007,0.008] | 0.007*** [0.006,0.008] | 0.006 [-0.001,0.013] | 0.006 [-0.002,0.013] |
| Sex | -0.013* [-0.024,-0.002] | -0.014* [-0.026,-0.003] | -0.030*** [-0.046,-0.015] | -0.024** [-0.040,-0.008] |
| White |  | 0.000 [ref] |  |  |
| Non-White |  | 0.042*** [0.023,0.060] |  |  |
| SEP - High |  | 0.000 [ref] |  | 0.000 [ref] |
| Intermediate |  | 0.002 [-0.009,0.012] |  | -0.007 [-0.024,0.010] |
| Low |  | -0.002 [-0.022,0.017] |  | -0.019 [-0.047,0.010] |
| Never smoker |  | 0.000 [ref] |  | 0.000 [ref] |
| Ex-smoker |  | 0.013* [0.003,0.023] |  | 0.010 [-0.007,0.027] |
| Current 1-10 |  | 0.035* [0.006,0.063] |  | 0.016 [-0.031,0.063] |
| Current 11+ |  | 0.010 [-0.017,0.036] |  | 0.037 [-0.000,0.074] |
| Constant | 0.760*** [0.752,0.768] | 0.750*** [0.740,0.761] | 0.661*** [0.634,0.688] | 0.651*** [0.621,0.682] |
| Observations | 4021 | 4011 | 1381 | 1252 |

95% confidence intervals in brackets

* p<0.05, ** p<0.01, *** p<0.001
